# Bioethics and artificial intelligence: between deliberation on values and rational choice theory

**DOI:** 10.3389/frobt.2023.1140901

**Published:** 2023-06-19

**Authors:** Boris Julián Pinto-Bustamante, Julián C. Riaño-Moreno, Hernando Augusto Clavijo-Montoya, María Alejandra Cárdenas-Galindo, Wilson David Campos-Figueredo

**Affiliations:** ^1^ Bioethics Department, Bioethics, Life Sciences Research Group, Universidad El Bosque, Bogotá, Colombia; ^2^ School of Medicine and Health Sciences, Universidad del Rosario, Medical and Health Sciences Education Research Group, Bogotá, Colombia; ^3^ Member of the Regional Committee Number 1—Donation and Transplantation Network, Bogotá, Colombia; ^4^ Faculty of Medicine, Cooperative University of Colombia, Villavicencio, Colombia; ^5^ Medical Subdirection, National Institute of Cancerology, Bogotá, Colombia; ^6^ Medical, Universidad El Bosque, Bogotá, Colombia

**Keywords:** echo chamber, artificial intelligence, filter bubble, decision-making, algorithmic reasoning, rational choice theory

## Abstract

The present work revisits how artificial intelligence, as technology and ideology, is based on the rational choice theory and the techno-liberal discourse, supported by large corporations and investment funds. Those that promote using different algorithmic processes (such as filter bubbles or echo chambers) create homogeneous and polarized spaces that reinforces people’s ethical, ideological, and political narratives. These mechanisms validate bubbles of choices as statements of fact and contravene the prerequisites for exercising deliberation in pluralistic societies, such as the distinction between data and values, the affirmation of reasonable dissent, and the relevance of diversity as a condition indispensable for democratic deliberation.

## 1 Introduction

In 1956, McCarthy described artificial intelligence (AI) as the simulation of human intelligence through an analog machine and digital machines (Ekmekci & Arda, 2020). Since then, a number of definitions have been proposed, and AI is now a thought to be a simulation of human behaviors like reasoning and thinking.

In this sense, AI can be perceived as a type of science or technology. In the first case, it is conceived as the body of knowledge focused on developing devices that mimic human behavior; it can be viewed as a technology as the group of devices created by humans (Coeckelbergh, 2020). For the purpose of this paper, AI will be viewed as a technology.

AI as a technology has the possibility of self-improvement, which takes the AI beyond the artifact category and is therefore partially subtracted from human control (Ekmekci & Arda, 2020). AI’s technology is transformed into an “ideology,” when the world is reduced to an analyzable dataset through the use of logic and arithmetic tools (Hui, 2020, 178), taking into account that machines are built for a certain vocation: “to tell the truth” (Sadín, 2020, p.17) (*aletheia*) through the attribution of human qualities (appreciation, evaluation, and decision, based on the maximizing rationality of the individual utility proposed by the Rational Choice Theory [RCT]) to synthetic processors which gives a “threatening power” (Sadín, 2020, p. 21) and an authoritarian power to the algorithmic reasoning of AI.

The convergence of artificial intelligence with various technologies such as big data, robotics, brain computer interfaces, and functional neuroimaging could configure systems capable of “mind-reading” or “dream-hacking,” even through brain spyware (Neuwirth, 2023). In light of these possibilities, the consolidation of neuro-rights as emerging rights is relevant (Yuste, 2017). While the debate persists regarding the current possibilities of “mind-reading” technologies based on functional magnetic resonance imaging associated with large language models (LLMs) (Reardon, 2023), the possibility of future optimization of these techniques, combined with other AI-based systems capable of subliminal manipulation and activation of human behavior (such as some neuromarketing techniques and recommendation systems), remain threats to human cognitive freedom (Neuwirth, 2023).

In this article, we argue RCT constitutes one of the theoretical and functional foundations for the development of artificial intelligence systems, which, by basing the efficiency of decisions on data modeling and the suppression of moral values, or their conversion into data in preference selection, can threaten cognitive freedom as well as deliberative practices regarding diverse moral values in societies with pluralistic aspirations through the formation of homogeneous and polarized interaction spaces (echo chambers) and algorithmic preference validation processes (filter bubbles).

## 2 AI and the RCT: the conversion of values into data

Human behavior and decision-making processes have been analyzed from various theoretical and experimental perspectives, which have been exploited for the development of artificial intelligence systems. One proposal states that human behavior in its different domains (sensory processing, motor control, decision-making, and learning) can be formulated in terms of probabilistic inference (Pouget et al., 2013).

Other studies propose the conjunction of Bayesian cognitive models with other models, such as fuzzy logic (which allows inference from modeling continuous, gradual, approximate, and imprecise data present in natural language) (Gentili, 2021), and judgment and decision-making (Chater et al., 2020), which describes the estimative processes agents use to choose from a set of available alternatives (Mishra, Novakowski & Gonzales, 2018). The latter approach is critical of classical RCT postulates, by questioning the notion of rational choice, the search for adaptive reasons in the presence of ambiguous rules, and the role of emotions in decision-making processes (Mellers, Schwartz & Cooke, 1998). Our hypothesis supports this critique.

RCT assumes concrete anthropology as a presupposition in its theoretical framework for the human behavioral sciences: the homo economicus as a selfish and magnifying archetype founded on rationality (parametric or strategic) (Ludolfo, 2005).

Since the middle of the 20th century, the RCT has been demonstrated as a theoretical body whose proposal is to address the understanding of human interactions based on economic models where collective action notions, class consciousness, and general interest are replaced by adding individual preferences in which the economic and political actor is an isolated individual with multiple resources, who has got the perfect information to choose rationally, to maximize the benefits and minimize the risk, despite the alternatives which are given by the market. With this aim in mind, in opposition to any notion of political or economic intervention of welfare, the market creates “a consistent and ostensible method of preferences aggregation” (Vidal de la Rosa, 2008). It is how individual interest and methodological individualism explain human motivations and social processes from utilitarianism and selfishness (Sen, 1986).

RCT, which has a solid foundation in probability math theory, claims that the agents tend to reduce the uncertainty as much as possible because there are some variables that escape from self-control. Individuals make use of prior knowledge to reduce this uncertainty, allowing for accurate prediction of future outcomes (Damasio, 1994). According to Kahneman (as cited in McElroy and Seta, 2003), the decision-making process is structured on two reasoning systems (System 1 [S1], or “intuitive,” and System 2 [S2], or “deliberate,”) used by subjects through preferences and cost–benefit assessment.

This dual-reasoning process has been analyzed in a variety of academic fields, including medicine. S1 is responsible for fast, intuitive, automatic, and cost-effective processing; it has lower conscious control, heuristic cognitive style, less analytic rigor, less reliable, lower predictive power, and is also highly susceptible to emotional influx and value biases (Croskerry et al., 2008).

S2 deals with a reasoning process whose cognitive style is systematic, analytical, slow, nearly inefficient in terms of cost, and has a higher predictive power, higher scientific rigor, and less exposure to emotional influx (Croskerry et al., 2008).

Our hypothesis affirms that AI is built on the RCT and the fusion of S1 and S2, where S1 become automatic and cost-effective but free of emotional valorization biases, while S2, which demands an ongoing deliberation around previous data and algorithmic processes that model the operative predictions, become a faster reasoning system, which decreases the likelihood of error (*hamartia*) (Kahneman and Tversky 1974). It turns values into facts (data), according to which the AI response algorithmic conform to axiological neutrality rule (Gracia, 2011).

Nevertheless, it is reasonable to assert that AI reductionism based on the suppression of value judgment and the emotional influx is incompatible, with both the evidence of the AI practices and human preferences and ethical principles of societies with pluralistic aspirations.

In the first place, the classic notion of the individual as a rational, self-centered, and maximizing *homo economicus* has been challenged by a more expansive view as a result to the contributions of social and cognitive sciences; these sciences explain a complex set of factors such as cultural, historical, environmental, institutional and neurobiological which module individual preferences, beyond the market and the utility. In the theories that attempt to explain both individual conduct and social behavior, the dimension of values (affections, emotions, feelings, beliefs) has taken a more prominent role. The traditional archetype of the *homo economicus* (selfish) coexists with the *homo reciprocans* (cooperative) as modes of social coexistence, recognized by heterogeneity, plasticity and versatility traits (Vidal de la Rosa, 2008). As Cortina affirms (2013), no human being can be deemed “amoral.”

Research evidence is consistent and proves the application of values biases (racial, genre, social class) present in AI programs, like facial recognition algorithms (Buolamwini y Gebru, 2018). These stereotypes correspond to the dissemination and amplification of negative value judgments prevalent in social, political, and cultural practices, which are expressed in terms of data representation that feeds AI information databases and automatic learning processes.

The reduction of values into the facts category constitutes a reversion to axiological objectivism of the dogma and doctrine, in which *facts* (accessible through sense perception) are equivalent to *values* (accessible through emotional/affective estimation) and, for the same reason, become universal, immutable, objective and trustworthy (Gracia, 2011). In this sense, algorithms depict a set of incontrovertible decision-making rules (uncritical/normative character).

RCT makes the assumption that rational choices are equal to choices made based on the premise of the “revealed preference,” in which some options are preferred over others, expressing an effective behavior and maximizing one’s own wellbeing. These preferences are given a numeric value that corresponds to their personal utility. Only a preference function can explain the relationship between the agent and the option.

The notion of preferences (in terms of consumption, navigation, views, and validation) results in assessments that are converted into data through the consistency test of the algorithmic reasoning (Gracia, 2019). According to Sen (1986), this *homo economicus* described by the RCT is a “rational fool”, incapable of functioning in social life.

Sen (1986) describes a tension between “ethic” preferences (what the subject believes is right from the perspective of social values) and “subjective” preferences (what the subject finds preferable from his or her personal interest view). From this, he postulates a principle of meta-sorting of preferences, in which functions of introspection (deliberation with oneself), communication (deliberation with others), and the virtue of the altruist commitment (as a feeling of duty with others which transcend the immediate consideration of the consequences) perform a fundamental role.

Finally, instead of creating a space for individual expression, the algorithmic personalization of preferences opposes the analytical, logical, and reflexive dimension of S2 because it limits the necessary conditions for discussion, debate, and deliberation regarding facts and values. We will analyze the filter bubble and echo chambers categories regarding this point.

### 2.1 Filter bubble and selective exposure

The term “filter bubble” was coined by Internet activist Eli Pariser (2011) in his text *The Filter Bubble: What the Internet Is Hiding from You*. It refers to the algorithmic customization effect that digital platforms or social media use networks offer. This idea relates to algorithms’ capability to adapt the user’s experiences; when people are part of a “bubble,” they are constantly exposed to information that matches their previous consumption behaviors (Spohr, 2017).

The algorithmic customization process is characterized by the following:

1. People are alone in their customized information bubbles.
2. The bubble is invisible; therefore, most of the time, people do not know what type of data is recollected and analyzed, leading to a wrong belief that information is not biased.
3. People do not actively choose to enter into the “filter bubble” (Sindermann et al., 2020) (even if empirical data contradict this affirmation) (Bakshy et al., 2015).

In this sense, filter bubbles are a reinforcing mechanism of “selective exposure”: the tendency of people to only be exposed to information that matches their interests, opinions, and beliefs while avoiding information that does not sympathize with or challenge their position (Spohr, 2017).

Therefore, it could enhance the biases relating to the fragmentary perspectives of the world (Pariser, 2011).

On the one hand, it lessens the likelihood of facing experiences, attitudes, and opposing opinions. On the other hand, it introduces confirmation biases, which are tendencies to search, select, and interpret information according to a particular belief system (Brugnoli et al., 2019), reinforcing preconceptions and prejudices. Additionally, it retracts the individual from negation (as understood by Hegel’s dialectic process) and supports the idea of *negation of the negation*, not as a transformative synthesis based on the confrontation of arguments but rather as the repudiation of one thesis over the other dissertation.

Algorithms function as groups of rules that validate preferences that are immune to critics (uncritical/legitimate character) and configure amplification spaces of their narratives, so-called *echo chambers* (Cinelli et al., 2021).

### 2.2 Echo chambers or negation of the conflict

According to Bakshy et al. (2015), filter bubbles create an algorithmic tunnel of data and information built by two mechanisms, a passive and an active: First, it develops an algorithmic selection that functions as a form of system preference validation (passive way). Then, it sets a simple operation of connecting and removing contacts and products on social media (active way), establishing preferential connections with groups and products among people who share the same core of interests and values *(echo chambers)*, resulting in cycles of self-affirmation, confirmation, and amplification that send off the difference and act as a “sounding board of the isolated Id” (Han, 2017) that fosters polarization (Bail et al., 2018).

Nevertheless, several authors like Bruns (2019) argue that there is an overestimation of this phenomenon and considers that the filter bubble concept is unreliable and lacks adequate empirical research.

The main cognitive mechanisms through which one’s own belief systems are reinforced in these homogeneous and polarized environments are *Conflict avoidance* (the tendency to avoid the confirmation of the own error) and the *Search for reinforcements* (the affirmation of the own beliefs) (Brugnoli et al., 2019). For instance, it is well known that *echo chambers*, although they emerge in virtual environments, they can spread quickly to their geographical proximity between groups of people who interact as offline echo chambers. It involved the opposition to the Brexit campaign (Bastos et al., 2018). It is now evident that its contents have a greater chance of going viral, which causes the spread of rumors, especially political ones (Choi et al., 2020).

The dynamics of the *echo chambers* have also been a topic studied by social sciences during the COVID-19 pandemic, for example, in relation to the COVID-19 vaccine (Burki, 2020). Despite being smaller, anti-vaccine movements have more interaction and have greater effect on social media than pro-vaccine movements do. In addition, the simulation models suggest that the anti-vaccine movement will be more prominent online (Johnson et al., 2020).

There is also a lot of concern in the education environment because *echo chambers* are fed from content loops favored by algorithm filters in social media (García-Bullé, 2019), designed to promote time consumption and the purchase of products and services. *Echo chambers* describe algorithms as rules that limit interaction and dissension (consensual/uncritical character).

## 3 Conclusion

Epstein et al. (2015) demonstrated that people’s voting preferences could be influenced, especially those who are very indecisive. Biases added to search engines and web search rankings affect votes by modifying how information is presented. It indicates that decision-making is compromised by undermining individual decision processes through the use of biased information provided by bubble filters, echo chambers and robust algorithmic systems.

As seen in the Facebook and Cambridge Analytica case, value expressions such as preferred reactions to various social media posts are converted into data that can be manipulated and used for market analyses and political trends (The Conversation, 2021). Moreover, the algorithmic, corporative, and political biopower turns values and emotions into data to model people’s private emotions and feelings (fear, anger, joy, indignation) for political and corporate purposes (Etxeberría, 2008, 23).

*Echo chambers* favor the “enclave deliberation” based on agents who share similar ideas where there is minimal room for disagreement (Bordonaba-Plou, 2019). In this case, there is a little chance of deliberation to achieve consensus and no room for reasonable dissension (Savulescu and Wilkinson, 2018), which can be understood as insufficient data, error, or maliciousness by the *echo chamber* dominant group (Lachlan et al., 2021, 23).

From this perspective, information technologies limit promises of democratization, so they restrict (both actively and passively) the interaction opportunities between those who share divergent values and attitudes. As part of what Gracia (2011) referred to as the “construction of values,” it is important to promote the public discussion around ethical, social, and political implications of the AI and the accountability of how databases and algorithmic processes that structure the digital system are configured, as well as to improve education to deliberate about virtual surroundings (Raphael, 1976; Bechmann and Nielbo, 2018; Andrew, 2019; Kaplan and Haenlein, 2019; Nechushtaia and Lewis, 2019).

## Data Availability

The original contributions presented in the study are included in the article/Supplementary Material, further inquiries can be directed to the corresponding author.

## References

[B1] Andrew N. (2019). IA para todos by deeplearning.ai. www.coursera.org.

[B2] Bail C. A. Argyle L. P. Brown T. W. Bumpus J. Chen H. Hunzaker F. (2018). Exposure to opposing views on social media can increase political polarization. Proc. Natl. Acad. Sci. U. S. A. 115 (37), 9216–9221. 10.1073/pnas.1804840115 30154168 PMC6140520

[B3] Bakshy E. Solomon M. Adamic L. (2015). Exposure to ideologically diverse news and opinion on Facebook. Science 348 (6239), 1130–1132. 10.1126/science.aaa1160 25953820

[B4] Bastos M. Mercea D. Baronchelli A. (2018). The geographic embedding of online echo chambers: Evidence from the Brexit campaign. PLoS ONE 13 (11), 02068411–e206916. 10.1371/journal.pone.0206841 PMC6214567 30388169

[B5] Bechmann A. Nielbo K. (2018). Are we exposed to the same “news” in the news feed? Digit. Journal. 6 (8), 990–1002. 10.1080/21670811.2018.1510741

[B6] Bordonaba-Plou D. (2019). Polarización como impermeabilidad: Cuando las razones ajenas no importan. Cinta Moebio 66, 295–309. 10.4067/s0717-554x2019000300295

[B7] Brugnoli E. Cinelli M. Walter Q. Antonio S. (2019). Recursive patterns in online echo chambers. Sci. Rep. 9 (1), 1–18. 10.1038/s41598-019-56191-7 31882591 PMC6934735

[B8] Bruns A. (2019). Are filter bubbles real? Cambridge, UK: Polity Press.

[B9] Buolamwini Joy Gebru Timnit (2018). Gender shades: Intersectional accuracy disparities in commercial gender classification. Proc. Mach. Learn. Res. 81, 1–15. http://proceedings.mlr.press/v81/buolamwini18a/buolamwini18a.pdf.

[B10] Burki T. (2020). The online anti-vaccine movement in the age of COVID-19. Lancet Digital Health 2 (10), e504–e505. 10.1016/s2589-7500(20)30227-2.-e505 32984795 PMC7508526

[B11] Chater N. Zhu J.-Q. Spicer J. Sundh J. León-Villagrá P. Sanborn M. (2020). Probabilistic biases meet the bayesian brain. Curr. Dir. Psychol. Sci. 29 (5), 506–512. 10.1177/0963721420954801

[B12] Choi D. Chun S. Oh H. Han J. Taekyoung Kwon T. (2020). Rumor propagation is amplified by echo chambers in social media. Sci. Rep. 10 (1), 310–10. 10.1038/s41598-019-57272-3 31941980 PMC6962360

[B13] Cinelli M. Morales G. F. Galeazzi A. Walter Q. Starnini M. (2021). The echo chamber effect on social media. Proc. Natl. Acad. Sci. U. S. A. 118 (9), e2023301118. 10.1073/pnas.2023301118 33622786 PMC7936330

[B14] Coeckelbergh M. (2020). AI ethics. Cambridge, MA, USA: The MIT Press.

[B15] Cortina A. (2013). Para qué sirve realmente la ética? Editor. Paid.

[B16] Croskerry P. Karen S. C. Stephen S. Robert W. (2008). Patient safety in emergency medicine. Philadelphia, PA, USA: Wolters Kluwer/ Lippincott Williams & Wilkins, 213–218. Chapter 31.

[B17] Damasio A. (1994). “Descartes' error (P. Jacomet, trad.; 3.a ed.),” in Editorial andrés bello. (Original work published 1994).

[B18] Ekmekci P. E. Berna A. (2020). Artificial intelligence and Bioethics. Switzerland, AG: Springer Briefs in Ethics. 10.1007/978-3-030-52448-7

[B19] Epstein R. Robertson R. E. (2015). The search engine manipulation effect (SEME) and its possible impact on the outcomes of elections. Proc. Natl. Acad. Sci. 12 (33), E4512–E4521. 10.1073/pnas.1419828112 PMC4547273 26243876

[B20] Etxeberría X. (2008). Por una ética de los sentimientos en el ámbito público. Bakeaz: Bilbao, Spain, 23.

[B21] García B. Sofía S. (2019). La cámara de eco y la amenaza al pensamiento crítico-humano. Observatorio I. para el futuro de la educación. https://observatorio.tec.mx/edu-news/camara-eco-pensamiento-critico.

[B22] Gentili P. L. (2021). Establishing a new link between fuzzy logic, neuroscience, and quantum mechanics through bayesian probability: Perspectives in artificial intelligence and unconventional computing. Molecules 26 (19), 5987. 10.3390/molecules26195987 34641530 PMC8512172

[B23] Gracia D. (2011). La cuestión del valor. Real Academia de Ciencias Morales y Políticas, Madrid, Spain, 162.

[B24] Gracia D. (2019). Bioética mínima. Triacastela, Spain, 66–107.

[B25] Han B. C. (2017). La expulsión de lo distinto. Herder.

[B26] Hui Y. (2020). Fragmentar el Futuro: Ensayos sobre Tecnodiversidad. Caja Negra.

[B27] Johnson N. F. Velásquez N. Restrepo N. J. Leahy R. Gabriel N. El Oud S. (2020). The online competition between pro- and anti-vaccination views. Nature 582 (7811), 230–233. 10.1038/s41586-020-2281-1 32499650

[B28] Kaplan A. Haenlein M. (2019). Siri, siri, in my hand: Who’s the fairest in the land? On the interpretations, illustrations, and implications of artificial intelligence. Bus. Horizons 62 (1), 15–25. 10.1016/j.bushor.2018.08.004

[B29] Lachlan K. A. Hutter E. Gilbert C. (2021). Covid-19 echo chambers: Examining the impact of conservative and liberal news sources on risk perception and response. Health Secur. 19 (1), 21–30. 10.1089/hs.2020.0176 33470883 PMC9195485

[B30] Ludolfo P. (2005). Teorías de la decisión racional y de la acción colectiva. Sociológica 20 (57), 13–34. https://https://www.redalyc.org/pdf/3050/305024871002.pdf.

[B31] McElroy T. Seta J. J. (2003). Framing effects: An analytic-holistic perspective. J. Exp. Soc. Psychol. 39, 610–617. Publicado por Elsevier (ISSN: 0022-1031). 10.1016/s0022-1031(03)00036-2

[B32] Mellers B. A. Schwartz A. Cooke A. (1998). Judgment and decision making. Annu. Rev. Psychol. 49 (1), 447–477. 10.1146/annurev.psych.49.1.447 9496629

[B33] Mishra S. Novakowski D. Gonzales J. (2018). “Judgment and decision-making,” in Encyclopedia of evolutionary psychological science. Editors Shackelford T. Weekes-Shackelford V. (Berlin, Germany: Springer). 10.1007/978-3-319-16999-6_628-1

[B34] Nechushtaia E. Lewis S. C. (2019). What kind of news gatekeepers do we want machines to be? Filter bubbles, fragmentation, and the normative dimensions of algorithmic recommendations. Comput. Hum. Behav. 90, 298–307. 10.1016/j.chb.2018.07.043

[B35] Neuwirth R. J. (2023). The EU artificial intelligence act: Regulating subliminal AI systems. London: Routledge.

[B36] Pariser E. (2011). The filter bubble. What the Internet is hiding from you. The Penguin Group. London, UK.

[B37] Pouget A. Beck J. M. Ma W. J. Latham P. E. (2013). Probabilistic brains: Knowns and unknowns. Nat. Neurosci. 16 (9), 1170–1178. 10.1038/nn.3495 23955561 PMC4487650

[B38] Raphael B. (1976). The thinking computer: Mind inside matter. W.H. Freeman and Company.

[B39] Reardon S. (2023). Mind-reading machines are here: Is it time to worry? Nature 617, 236. Advance online publication. 10.1038/d41586-023-01486-z 37130904

[B40] Sadín É. (2020). La inteligencia Artificial o el Desafío del Siglo: Anatomía de un Antihumanismo Radical. Caja Negra.

[B41] Sen A. (1986). Los tontos racionales: Una crítica de los fundamentos conductistas de la teoría económica. Filos. Teoría Económica, Frank Hahn Martina Hollis, 172–217. Fondo de Cultura Económica.

[B42] Sindermann C. Elhai J. D. Moshagen M. Montag C. (2020). Age, gender, personality, ideological attitudes and individual differences in a person's news spectrum: How many and who might be prone to “filter bubbles” and “echo chambers” online? Heliyon 6 (1), e03214. 10.1016/j.heliyon.2020.e03214 32051860 PMC7002846

[B43] Spohr D. (2017). Fake news and ideological polarization: Filter bubbles and selective exposure on social media. Bus. Inf. Rev. 34 (3), 150–160. 10.1177/0266382117722446

[B44] The Conversation (2021). Targeted ads isolate and divide us even when they’re not political – new research. https://theconversation.com/targeted-ads-isolate-and-divide-us-even-when-theyre- not-political-new-research-163669.

[B45] Vidal D. L. R. Godofredo G. (2008). La teoría de la decisión racional en las ciencias sociales. Sociológica 23 (67), 221–236. http://www.scielo.org.mx/pdf/soc/v23n67/v23n67a9.pdf.

[B46] Wilkinson D. Savulescu J. (2018). Ethics, conflict and medical treatment for children: From disagreement to dissensus. Elsevier. Amsterdam, Netherlands. [Internet]. 30835404

[B47] Yuste R. Goering S. Bi G. Carmena J. M. Carter A. Fins J. J. (2017). Four ethical priorities for neurotechnologies and AI. Nat. News 551, 159–163. 10.1038/551159a PMC8021272 29120438

