# Corrigendum: Bioethics and artificial intelligence: between deliberation on values and rational choice theory

**DOI:** 10.3389/frobt.2023.1251568

**Published:** 2023-07-18

**Authors:** Boris Julián Pinto-Bustamante, Julián C. Riaño-Moreno, Hernando Augusto Clavijo-Montoya, María Alejandra Cárdenas-Galindo, Wilson David Campos-Figueredo

**Affiliations:** ^1^ Bioethics Department, Bioethics, Life Sciences Research Group, Universidad El Bosque, Bogotá, Colombia; ^2^ School of Medicine and Health Sciences, Universidad del Rosario, Medical and Health Sciences Education Research Group, Bogotá, Colombia; ^3^ Member of the Regional Committee Number 1—Donation and Transplantation Network, Bogotá, Colombia; ^4^ Faculty of Medicine, Cooperative University of Colombia, Villavicencio, Colombia; ^5^ Medical Subdirection, National Institute of Cancerology, Bogotá, Colombia; ^6^ Medical, Universidad El Bosque, Bogotá, Colombia

**Keywords:** echo chamber, artificial intelligence, filter bubble, decision-making, algorithmic reasoning, rational choice theory

In the published article, there was an error in **affiliation** 1. Instead of “Bioethics Department, El Bosque University, Bogotá, Colombia”, it should be “Bioethics Department, Bioethics, Life Sciences Research Group, Universidad El Bosque, Bogotá, Colombia”.

In the published article, there was an error in **affiliation** 2. Instead of “School of Medicine and Health Sciences, Del Rosario University, Bogotá, Colombia”, it should be “School of Medicine and Health Sciences, Universidad del Rosario, Medical and Health Sciences Education Research Group, Bogotá, Colombia”.

In the published article, there was an error in **affiliation** 5. Instead of “Department of Bioethics, El Bosque University, Bogotá, Colombia”, it should be “Bioethics Department, Bioethics, Life Sciences Research Group, Universidad El Bosque, Bogotá, Colombia”.

In the published article, there was an error in **affiliation** 7. Instead of “Medical, El Bosque University, Bogotá, Colombia”, it should be “Medical, Universidad El Bosque, Bogotá, Colombia”.

In the published article, there was an error. error in the translation of a sentence: “In this sense, algorithms can be a part of groups of incontrovertible decision-making rules (uncritical/normative character)”.

A correction has been made to **2 AI and the RCT: the conversion of values into data**, paragraph 12. This sentence previously stated:

“In this sense, algorithms can be a part of groups of incontrovertible decision-making rules (uncritical/normative character.”

The corrected sentence appears below:

“In this sense, algorithms depict a set of incontrovertible decision-making rules (uncritical/normative character).”

The authors apologize for this error and state that this does not change the scientific conclusions of the article in any way. The original article has been updated.

